# Pilot Project for a Web-Based Dynamic Nomogram to Predict Survival 1 Year After Hip Fracture Surgery: Retrospective Observational Study

**DOI:** 10.2196/34096

**Published:** 2022-03-30

**Authors:** Graeme McLeod, Iain Kennedy, Eilidh Simpson, Judith Joss, Katriona Goldmann

**Affiliations:** 1 Department of Anaesthesia Ninewells Hospital National Health Service Tayside Dundee United Kingdom; 2 School of Medicine University of Dundee Ninewells Hospital Dundee United Kingdom; 3 Crosshouse Hospital National Health Service Ayrshire and Arran Kilmarnock United Kingdom; 4 William Harvey Research Institute Barts and the London School of Medicine & Dentistry London United Kingdom

**Keywords:** hip fracture, survival, prediction, nomogram, web, surgery, postoperative, machine learning, model, survival, mortality, hip, fracture

## Abstract

**Background:**

Hip fracture is associated with high mortality. Identification of individual risk informs anesthetic and surgical decision-making and can reduce the risk of death. However, interpreting mathematical models and applying them in clinical practice can be difficult. There is a need to simplify risk indices for clinicians and laypeople alike.

**Objective:**

Our primary objective was to develop a web-based nomogram for prediction of survival up to 365 days after hip fracture surgery.

**Methods:**

We collected data from 329 patients. Our variables included sex; age; BMI; white cell count; levels of lactate, creatinine, hemoglobin, and C-reactive protein; physical status according to the American Society of Anesthesiologists Physical Status Classification System; socioeconomic status; duration of surgery; total time in the operating room; side of surgery; and procedure urgency. Thereafter, we internally calibrated and validated a Cox proportional hazards model of survival 365 days after hip fracture surgery; logistic regression models of survival 30, 120, and 365 days after surgery; and a binomial model. To present the models on a laptop, tablet, or mobile phone in a user-friendly way, we built an app using Shiny (RStudio). The app showed a drop-down box for model selection and horizontal sliders for data entry, model summaries, and prediction and survival plots. A slider represented patient follow-up over 365 days.

**Results:**

Of the 329 patients, 24 (7.3%) died within 30 days of surgery, 65 (19.8%) within 120 days, and 94 (28.6%) within 365 days. In all models, the independent predictors of mortality were age, BMI, creatinine level, and lactate level. The logistic model also incorporated white cell count as a predictor. The Cox proportional hazards model showed that mortality differed as follows: age 80 vs 60 years had a hazard ratio (HR) of 0.6 (95% CI 0.3-1.1), a plasma lactate level of 2 vs 1 mmol/L had an HR of 2.4 (95% CI 1.5-3.9), and a plasma creatinine level of 60 vs 90 mol/L had an HR of 2.3 (95% CI 1.3-3.9).

**Conclusions:**

In conclusion, we provide an easy-to-read web-based nomogram that predicts survival up to 365 days after hip fracture. The Cox proportional hazards model and logistic models showed good discrimination, with concordance index values of 0.732 and 0.781, respectively.

## Introduction

As many as 7 of 100 patients die in the first 30 days after hip fracture [1-4]. Mortality in the first 365 days after hip fracture surgery varies between 14% and 23% of patients [5,6]. Identification of individual risk can inform anesthetic and surgical decision-making and potentially improve outcomes. However, mathematical models can be complex and difficult to interpret, and the effect of changes in continuous or categorical variables may not be obvious. Graphical presentation of data is a pivotal technique in science and key to better communication [7]. Nomograms present covariables in a relatively easy-to-understand way and are commonly used to inform clinicians and patients of the risk of mortality in prostate cancer [8]. However, interpreting predictive indices and applying them to individual patients is difficult [9]. Apps based on the R package (RStudio v 1.3.1093; R Foundation for Statistical Computing), such as Shiny, can be used to translate statistical models into easy-to-understand, web-based interactive nomograms that readily demonstrate differences between low-risk and high-risk patients. One example is the DynNom package [7] in R that predisplays the results of statistical models as a dynamic nomogram and readily allows individual prediction with 95% CI.

Anesthetic guidelines and protocols increasingly drive standardization of practice [10]. However, we believe that individual identification of risk is more likely to improve outcomes [11]. Several risk-specific and generic surgical risk indices are available that predict mortality after hip fracture surgery, but most are limited to prediction of mortality 30 days after operation. These indices discriminate well but lack adequate calibration [12]. The Nottingham Hip Fracture Score has been validated nationally [2] and internationally [13] and is commonly used, but its 365-day score discriminates only between low- and high-risk patients. Moreover, the more complex a statistical model is due to nonlinearity and interactions, the more difficult it is to comprehend and apply. As such, survival models based on time-to-death data [14] are uncommon. Therefore, there is a need to develop an easy-to-interpret app for time-to-event as well as binary-outcome data. Proving such an app's utility locally would provide a platform for prospective development of a large multicenter database that could inform a statistical model with high calibration and discrimination. This model could easily be used at the bedside with a laptop, tablet, or mobile phone in order to inform staff and laypeople of outcomes after hip fracture surgery. Therefore, our primary objective was to develop a web-based nomogram from clinical data collected over an 8-month period from patients undergoing hip fracture surgery at a single tertiary center.

## Methods

We conducted a retrospective study of patients undergoing hip fracture surgery. Our study included data collection, statistical modeling, and app development.

### Data Collection

We collected preoperative and operative data from all patients presenting for hip fracture surgery at Ninewells Hospital, Dundee, Scotland, over an 8-month period between May 1, 2016, and December 31, 2016. The patients’ case notes, anesthetic charts, and operative notes for the first year after surgery were reviewed as part of a fourth-year medical student project.

The data included patient characteristics, comorbidities, and health status. Patient characteristics recorded on admission included age, sex, BMI, fracture side (left or right), type of fracture (intracapsular or extracapsular), their type of residence before the fracture, and a social deprivation score based on the Scottish Index of Multiple Deprivation 2016 (SIMD16) database, which measures deprivation in 6976 residential areas in Scotland [15]. We used the SIMD16 vigintile database, which ranks deprivation from 1 (the most deprived residential areas) to 20 (the least deprived residential areas). Blood tests were taken on hospital admission and included white cell count and levels of hemoglobin, creatinine, lactate, and C-reactive protein. With regard to surgery, we noted the physical status of the patient according to the American Society of Anesthesiologists (ASA) Physical Status Classification System, the type of anesthesia (general or spinal), the type of surgical implant, and the time the operation took place. Operations performed between 9 AM and 5 PM were classified as daytime operations, those performed between 5 PM and 10 PM as evening operations, and those performed between 5 PM and 9 AM as nighttime operations. Postoperatively, we noted the need for transfusion, presence of acute kidney injury, cardiovascular complications such as pulmonary embolus or myocardial infarction, and infection from any source (wound, urinary, or respiratory). We recorded the date of hospital discharge and the destination of the patient. The type of residence of the patient before the fracture and upon discharge from the ward were classified as the following: home (either the patient’s own home or sheltered housing), care home, acute-care hospital, rehabilitation hospital, or long-term-care hospital. Our primary outcome was time to death by any cause within 365 days of hip fracture surgery.

### Model Development

We developed 4 statistical models: a global Cox proportional hazards model using all available covariates, a final Cox proportional hazards model, a generalized linear model, and a logistic regression model. Models and nomograms were developed using the R packages “shiny,” “ggplot2,” “ggpub,” “stargazer,” “rms,” “shinythemes,” and “plotly.”

Our modeling strategy was based on that recommended by Harrell and Steyerberg [9]. We selected variables based on our clinical experience and evidence from published studies. We collected as much pertinent data as possible, with wide distributions for predictor values. We hypothesized that continuous variables were nonlinear. We used imputation to replace missing covariables with the median value. We restricted the number of events per variable in the model according to the following equation: events per variable = events or outcomes/15. We prespecified the complexity of the model and initially allotted 3 cubic splines (knots) to continuous variables in order to detect any nonlinear relationships between variables and outcomes and allotted 1 *df* to categorical data.

We first created a global model using all variables and tested the association of each predictor with outcomes adjusted for all other predictors and the *df* used. We reduced the model by calculating the *df* that could be spent and deciding how they should be spent. We ranked the apparent importance of predictors of death by plotting the Akaike information criterion, defined as *χ*^2^ – 2 *df*. Initial estimation of shrinkage (γ) needed used the formula γ = (*χ*^2^ – p)/ *χ*^2^). We also interpreted the model graphically and decided which parameters should be retained for bootstrap validation of calibration and discrimination. Continuous variables that showed a linear relationship with outcome were restricted to 1 *df*.

Overfitting and effects of shrinkage were assessed using the corrected calibration slope. This was obtained using bootstrapping bias–corrected (overfitting minus corrected) estimates of predicted vs observed values. In order to check proportional hazards assumptions, we examined scaled Schoenfeld residuals.

### Model Validation

Prediction errors were assessed using the log-likelihood ratio (*χ*^2^) for continuous data and the Brier score for binary data. The ability to discriminate between low-risk and high-risk patients was measured with *R*^2^, the Gini index from 0 to 1, a robust measure of variation, and measures of rank discrimination, such as the *C* index and Somers Dxy. The *C* index represents the probability of concordance, *C*, between predicted and observed survival, and is equivalent to the area under the receiver operator characteristics curve (AUROC). Concordance is defined as the proportion of all pairs of subjects whose survival time can be ordered such that the subject with the higher predicted survival is the one who survived longer. Dxy is the difference between concordance and discordance probabilities and relates to the *C* index by the equation Dxy = 2(*C* − 0.5). Internal calibration and validation used the bootstrap.

### Model Comparisons

Our secondary objectives were to develop a 365-day logistic regression model and a 365-day generalized linear model for binomial response data for sensitivity analysis, and to develop additional 30-day and 120-day logistic models in order to compare accuracy against the routinely used Nottingham Hip Fracture Score.

### App Development

A data scientist (KG) developed an app using Shiny, a package from RStudio that builds interactive web applications with R. We created 3 files: ui.R to define the user interface A; server.R to interrogate data from the user interface and define the app logic; and functions.R to combine these 2 files and create the Shiny application.

The user interface (ui.R) consisted of a title, side panel, and main panel. The side panel contained a drop-down box with 4 models: the global Cox proportional hazards model, the final Cox proportional hazards model, the generalized linear model, and the logistic regression model. The side panel also had sliders for input of continuous variables over their range of values and follow-up time (0 to 365 days). The main panel consisted of 3 tabs: a prediction plot, a survival plot, and a model summary.

Prediction plots were displayed on a graph with probability on the x-axis. The mean was displayed as a colored square with horizontal lines representing the 95% CI for the outcome. Survival models showed a Kaplan-Meier plot of estimated survival probability over time. The app can be viewed at our page on the shinyapps website [16].

### Statistical Analysis

Continuous variables are presented as the mean (SD) and were analyzed using the Aspin-Welch unequal variance test. Nonparametric data were presented as the median (IQR, full range) and analyzed using the Mann-Whitney *U* test. Cross-tabulation of categorical data count (n) was analyzed using the *χ*^2^ test. Calculation of the AUROC for Nottingham Hip Fracture Score used GraphPad Prism 9 (GraphPad).

### Ethics Approval

Caldicott guardian approval was obtained from the University of Dundee on October 16, 2016. In the United Kingdom, Caldicott guardians provide ethical approval for interrogation of anonymous clinical databases.

## Results

### Data Collection

We recorded data from 329 patients, of whom 224 (68%) were female and 85 (32%) were male. We found that 4% of biochemical data were missing and replaced them with the median value. Over two-thirds of patients (224/329, 68%) were classified as ASA category III or IV. These categories indicate severe systemic disease and disease that is a constant threat to life, respectively. We found that 24 (7.3%) patients died within 30 days, 65 (19.8%) within 120 days, and 94 (28.6%) within 365 days of surgery. Patient characteristics, categorized according to survival or death within 365 days, are shown in Table 1.

**Table 1:** Characteristics of surviving and deceased patients 365 days after hip fracture surgery.

Variable			Surviving (n=235)		Deceased (n=94)		Difference (95% CI), odds ratio (95% CI)			*P* value	
Age in years, mean (SD)			82.5 (10.0)		80.9 (9.6)		1.5 (0.8 to 3.9)			.21	
**Sex, n (%)**								1.0 (0.6 to 1.7)			.94
	Male	61 (26)		24 (25.5)							
	Female	174 (74)		70 (74.5)							
BMI in kg/m^2^, mean (SD)			24.2 (5.7)		21.8 (4.2)		2.4 (0.9 to 3.8)			.002	
**Status according to American Society of Anesthesiologists Physical Status Classification System, n (%)**								N/A^a^			<.001
	I	7 (3)		0 (0)							
	II	62 (26.4)		5 (5.3)							
	III	113 (48.1)		58 (61.7)							
	IV	27 (11.5)		26 (27.7)							
**Type of residence, n (%)**								N/A			<.001
	Home	189 (80.4)		46 (48.9)							
	Care home	41 (17.4)		45 (47.9)							
	Rehabilitation hospital	3 (1.3)		3 (3.2)							
	Acute-care hospital	1 (0.4)		0 (0)							
	Long-term–care hospital	1 (0.4)		0 (0)							
Scottish Index of Multiple Deprivation 2016 score, median (IQR, full range)			11 (6 to 16, 1 to 20)		12 (8 to 16, 1 to 20)		1.0 (–1.0 to 2.0)			.42	
Stay in days, mean (SD)			12.6 (10.2)		12.1 (8.2)		0.5 (–1.7 to 2.6)			.67	
**Side, n (%)**								1.0 (0.6 to 0.6)			.89
	Left	123 (52.3)		50 (53.2)							
	Right	112 (47.7)		44 (46.8)							
**Implant type, n (%)**								N/A			.70
	Bipolar	18 (7.7)		3 (3.2)							
	Compression hip screw	80 (34)		35 (37.2)							
	Collarless, polished, tapered	26 (11.1)		1 (1.1)							
	Thompson	88 (37.4)		45 (47.9)							
	Femoral nail	23 (9.8)		10 (10.6)							
Hemoglobin in g/L, mean (SD)			120.3 (18.0)		115.6 (15.0)		4.7 (0.8 to 8.6)			.01	
White cell count in 10^9^/L, mean (SD)			11.8 (6.1)		11.1 (3.2)		0.8 (–0.2 to 1.8)			.14	
C-reactive protein in mg/L, median (IQR, full range)			6 (3 to 25, 2 to 299.0)		13 (3 to 46, 3 to 273)		1.0 (0.0 to 3.0)			.046	
Lactate in mmol/L, mean (SD)			1.47 (0.74)		1.70 (0.92)		0.24 (0.0 to 0.48)			.04	
Creatinine in µmol/L, mean (SD)			71.1 (27.5)		89.2 (42.0)		18.2 (8.4 to 27.8)			<.001	
**Operation time (%)**								N/A			.02
	Daytime (9 AM to 5 PM)	196 (83.4)		78 (83)							
	Evening (5 PM to 10 PM)	37 (15.7)		14 (14.9)							
	Night (10 PM to 9 AM)	2 (0.9)		2 (2.1)							
**Type of residence after discharge (%)**								N/A			<.001
	Home	100 (42.6)		13 (13.8)							
	Care home	61 (26)		42 (44.7)							
	Rehabilitation setting	54 (23)		18 (19.1)							
	Acute-care hospital	15 (6.4)		6 (6.4)							
	Long-term-care hospital	7 (3)		3 (3.2)							
	Died in hospital	2 (0.9)		8 (8.5)							

^a^N/A: not applicable.

### Model Development

**Figure 1:**
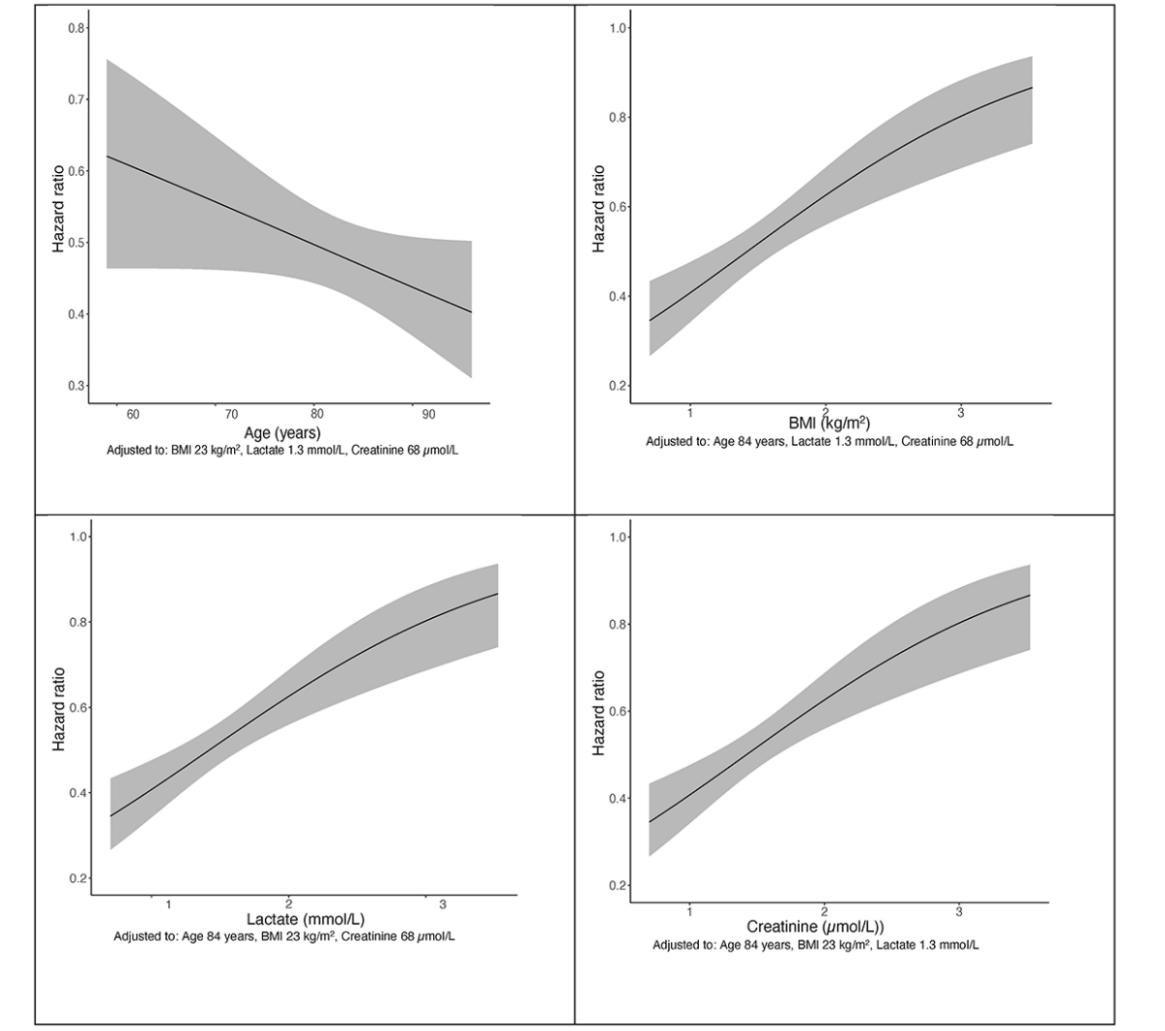
Final Cox proportional hazards model 365 days after hip fracture surgery. Hazard ratios show a reduced risk of death with increasing age and lower BMI. Risk of death rose with increased creatinine and lactate levels. Note the nonlinear increase in risk with creatinine level, and the increase in risk from values immediately above the physiological range.

A global Cox proportional hazards model using all covariates, a final Cox proportional hazards model, logistic regression models, and a generalized linear model were constructed from the data. The global Cox proportional hazards model took all covariates into account, whereas the final validated model was built within the statistical constraints discussed in the methods. The graphs in Figure 1 show continuous relationships between covariates and the probability of death using the final Cox proportional hazards model. The nonlinear relationship between creatinine and the risk of death (ie, HR) was calculated using cubic splines (Figure 1).

Independent predictors of mortality in the final Cox proportional hazards model included increased age, BMI, creatinine, lactate, and the combination of these factors. Examples of differences in mortality on admission included the following: age 80 vs 60 years had an HR of 0.6 (95% CI 0.3-1.1), a plasma lactate level of 2 vs 1 mmol/L had an HR of 2.4 (95% CI 1.5-3.9), and a plasma creatinine level 60 vs 90 µmol/L had an HR of 2.3 (95% CI 1.3-3.9).

### Model Validation

Validation results for the global and final Cox proportional hazards models are shown in Table 2.

**Table 2:** Model validation. Global and final Cox proportional hazards models. The final model was developed after iterative data reduction and calibration using bootstrap and showed good validation in 329 patients.

Model	*R^2a^*	LR (*χ*^2^)^b^	*P* value	Dxy^c^	C index^d^	g^e^
Global Cox proportional hazards model 365 days after surgery	0.364	*χ*^2^_22_=45.328	.002	0.623	0.812	1.897
Final Cox proportional hazards model 365 days after surgery	0.231	*χ*^2^_7_=43.113	<.01	0.474	0.732	1.360

^a^*R*^2^ coefficient of determination.

^b^Likelihood ratio chi-square test.

^c^Somers Dxy test.

^d^Concordance index.

^e^Gini index.

### Model Comparisons

The predictive variables identified using the final Cox proportional hazards model were similar to the predictive variables identified using the 365-day logistic regression and 365-day binomial models (Table 3).

Validation results for our secondary outcomes and the 30, 120, and 365-day logistic regression models are presented in Table 4.

Using our data, we calculated the AUROC for Nottingham Hip Fracture Score to be <0.61 (95% CI) at all time points (Table 5).

**Figure 2:**
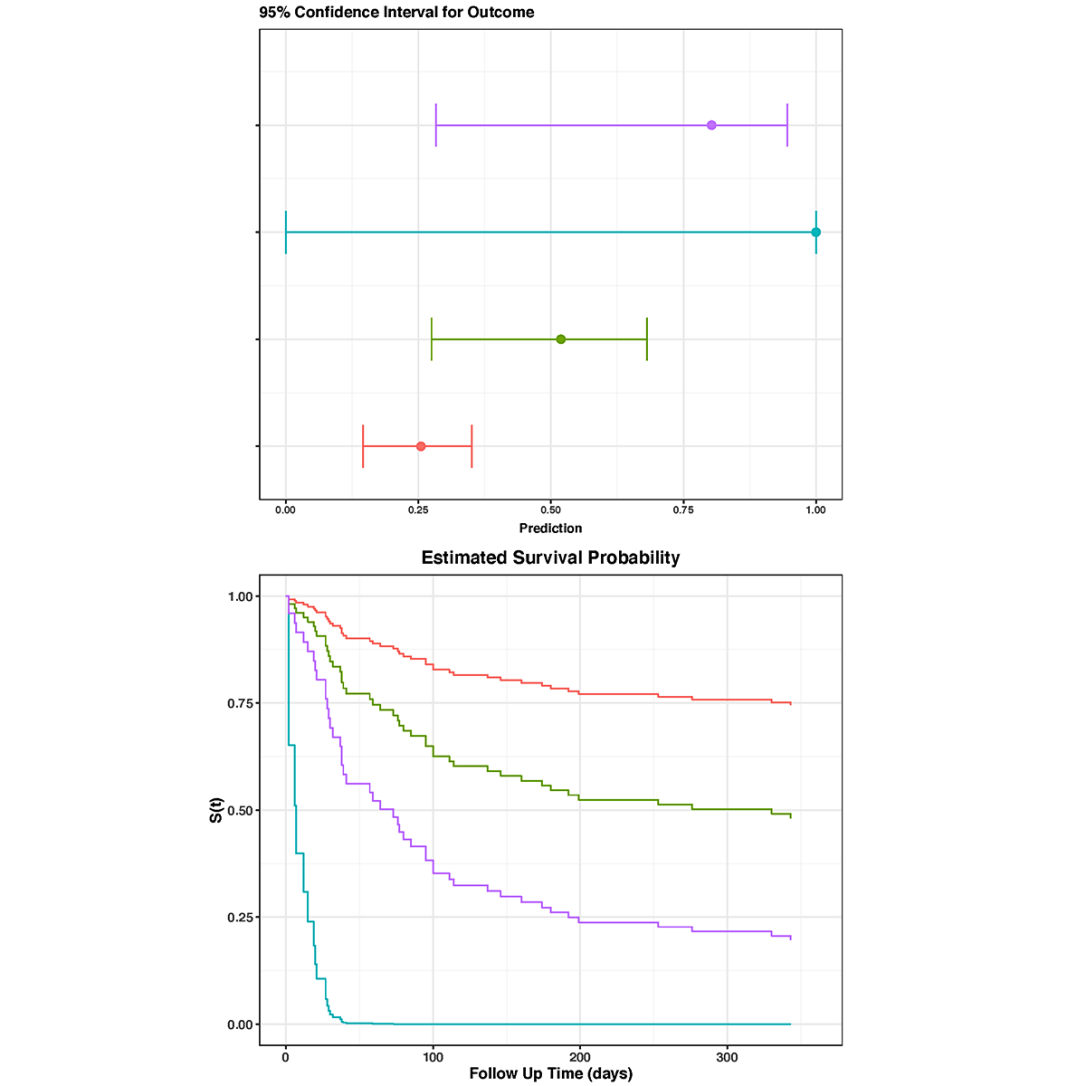
Dynamic nomogram. Top: sliders that are used to enter data for age (standardized to 80 years) and white cell count (standardized to 10 x 109/L). Bottom: 4 imaginary scenarios, differing in BMI, creatinine, and lactate. The red, green, blue, and purple lines represent the following values for BMI, creatinine, and lactate, respectively: 25 kg/m^2^, 80 µmol/L, 1.5; 15 kg/m^2^, 80 µmol/L, 1.5 mmol/L; 15 kg/m^2^, 80 µmol/L, 4 mmol/L; and 15 kg/m^2^, 140 µmol/L, 4 mmol/L. The dynamic nomogram is available on our website [16].

An example of an easy-to-interpret dynamic nomogram is presented in Figure 2. The variables of this nomogram can be altered using sliders. The digital nomogram is available online at our website [16].

**Table 3:** Independent variables predicting mortality in the final Cox proportional hazards model, a logistic model, and a binomial model. All models are 365 days after hip fracture surgery. Variables common to all models included age, BMI, lactate, and creatinine. Apostrophes indicate nonlinear restricted cubic splines.

Dependent variable	Final Cox proportional hazards model, regression coefficient (95% CI)	Logistic regression model, regression coefficient (95% CI)	Binomial model, regression coefficient (95% CI)
Age	0.976 (0.947 to 1.007)	–0.023 (–0.062 to 0.016)	–0.018 (–0.056 to 0.020)
BMI	0.913 (0.862 to 0.967)	–0.115 (–0.199 to –0.032)	–0.126 (–0.205 to –0.047)
White cell count	N/A^a^	0.138 (–0.109 to 0.385)	–0.028 (–0.105 to 0.048)
White cell count’	N/A	–0.196 (–0.453 to 0.062)	N/A
Lactate	0.003 (<0.001 to 0.199)	–5.519 (–10.812 to –0.226)	–0.899 (–0.095 to 1.893)
Creatinine	0.906 (0.817 to 1.005)	–0.072 (–0.198 to 0.055)	–0.031 (0.008 to 0.055)
Creatinine’	1.185 (1.030 to 1.364)	0.133 (–0.042 to 0.308)	N/A
Lactate*Creatinine	1.110 (1.037 to 1.189)	0.098 (0.013 to 0.183)	-0.007 (–0.018 to 0.004)
Lactate*Creatinine’	0.865 (0.788 to 0.951)	–0.134 (–0.250 to –0.018)	N/A
Constant	N/A	5.471 (–3.329 to 14.270)	0.491 (–3.379 to 4.360)

^a^N/A: not applicable.

**Table 4:** Logistic regression validation results.

Model	*R^2a^*	LR (*χ*^2^_9_)^b^	*P* value	Brier	Dxy^c^	C index^d^	g^e^
Logistic model (30 days)	0.714	17.390	.004	0.069	0.541	0.770	1.348
Logistic model (120 days)	0.396	21.280	.002	0.114	0.706	0.853	2.051
Logistic model (365 days)	0.277	37.252	<.001	0.147	0.562	0.781	1.619

^a^*R*^2^ coefficient of determination.

^b^Likelihood ratio chi-square test.

^c^Somers Dxy test.

^d^Concordance index.

^e^Gini index.

**Table 5:** Diagnostic results for Nottingham Hip Fracture Score.

Time	Area under the receiver operating characteristics curve (95% CI)	*P* value
30 days	0.576 (0.454-0.698)	.22
120 days	0.606 (0.538-0.674)	.003
365 days	0.602 (0.526-0.678)	.01

## Discussion

### Principal Findings

We provide proof of concept of a simple, dynamic digital nomogram created in R and Shiny that shows individual survival with the 95% CI after hip fracture surgery. The nomogram offers an easy, intuitive means of interpreting complicated models. Our models showed good discrimination and calibration. Lactate, creatinine, age, and BMI emerged as important predictors of mortality in all models.

### Comparison to Prior Work

Our data are consistent with previous studies demonstrating an association between higher serum lactate and mortality following hip fracture [18-20]. For example, we found that a rise in plasma lactate from 2 to 3 mmol/L increased the hazard ratio by >25%. Unlike previous studies, we did not arbitrarily define raised lactate as a level >2.5 mmol/L [21] or 3.0 mmol/L [18,20]. In fact, our nonlinear modeling of continuous lactate data showed an early, steep rise in the risk of mortality from 1 mmol/L. This has implications for clinical practice. It suggests that a lower-than-anticipated lactate level has an impact on short-term and long-term mortality and the need for early resuscitation. However, we are not aware of any randomized controlled trials that have examined fluid resuscitation in patients presenting with hip fracture. An association between prolonged lactate clearance and mortality may occur in the surgical intensive care unit population [22], but this cannot be extrapolated to the management of elderly patients with hip fracture.

Nonlinear modeling of our creatinine data also showed an early, steep rise in the risk of mortality. For example, a rise in plasma creatinine from 60 to 90 µmol/L more than doubled the risk of death. Once more, this demonstrates that changes just outside the normal physiological range may profoundly impact outcomes; clinicians should take note of such changes, rather than wait for grossly deranged blood results.

Our models also revealed that there was an inverse association of outcome with BMI [23,24] and that frailty and muscle mass had a significant long-term negative impact on survival after hip fracture surgery. For example, a reduction in BMI from 25 to 20 kg/m^2^ increased the hazard ratio by approximately one-third. Unlike other studies, we failed to show a significant effect of anemia. This probably reflects changes in patient blood management strategies since initial studies into this association were published [2,3]. Surprisingly, we showed an inverse relationship between age and outcome, in contrast to many other models [12,25,26]. This reflects increased comorbidities in our younger population, limiting the applicability of our model to other populations. Nevertheless, for comparison, we applied the Nottingham Hip Fracture Score to our data. Surprisingly, the Nottingham score showed much poorer discrimination compared to our dataset, with an AUROC <0.61 and a lack of statistical significance for 30-day predictions of mortality.

### Strengths and Limitations

Our study had 3 key strengths. First, rather than just focus on 30-day mortality, we observed our patients for 12 months in order to obtain a detailed temporal overview of outcomes after hip fracture surgery. Most models, in contrast, focus on measurement of 30-day mortality [4,7,9-12] and tend to reflect events during hospital stay. By contrast, the Nottingham Hip Fracture Score predicts 1-year mortality [13], but it divides patients according to a binary low risk/high risk classification based on a cut-off score.

Second, we used modeling techniques available in R. The nonlinearity of creatinine and the interaction with lactate justified our application of restricted cubic splines to continuous data. Although this allocated 3 degrees of freedom to continuous variables, this technique improved the accuracy of the model. We also used bootstrapping to validate our model. The advantage of bootstrapping is that the entire dataset can be used, unlike data splitting, which reduces the sample size for both model development and testing. Variable selection or stopping rules were not used, because these methods provide regression coefficients that are too high and confidence intervals that are too small. Neural networks, such as support vector machines, naive Bayes classifiers, and random forest classifiers, have been applied to hip fracture data sets, but were no better than logistic regression in predicting outcomes after surgery [27].

Third, our mortality was in line with national data. Mortality increased from 7.3% at 30 days to 28.6% at 365 days and allowed us to incorporate 5 variables with good calibration and validation.

A limitation of this study was insufficient data; we could not generate a model that incorporated all potential confounders. We suggest investigators capture data from the dimensions of risk recommended by Iezzoni [28], as these are most likely to explain variations in mortality. Such variables should not only include patient characteristics, recent health status, mental acuity, and quality of life, but also markers of acute clinical stability.

### Future Directions

We present an example of our dynamic nomogram online [16] but emphasize that, based on our global model, predictions can be improved by recruiting more patients. While this study identifies important risk factors for mortality in hip fracture patients and robustly demonstrates a proof of concept for an app-based dynamic nomogram of individualized mortality risk, medical apps in the United Kingdom must be registered with the Medicines and Healthcare Products Regulatory Agency as Class 1 medical devices prior to any clinical use, which requires prospective registration of data gathering. Our app is not registered and should not be used to guide specific patient treatment; the prototype app provided online is for educational purposes and to inform future research. As such, validation against a larger patient population is needed to validate the model and support a future application for Medicines and Healthcare Products Regulatory Agency device registration.

### Conclusion

We developed a dynamic nomogram for prediction of survival using Shiny that presents a Cox proportional hazards model and logistic and binomial models in an easy, intuitive, and interpretable format. All models identified lactate and creatinine levels at admission as independent predictors of mortality. Although our relatively small numbers limit external application at this time, our findings nevertheless show that acute hemodynamic changes drive mortality not just in the first 30 days, but also up to 1 year after operation.

## Abbreviations

ASA: American Society of Anesthesiologists
AUROC: area under the receiver operator characteristics curve
HR: hazard ratio
SIMD16: Scottish Index of Multiple Deprivation 2016
